# Impact of delayed mobile medical team dispatch for respiratory distress calls: a propensity score matched study from a French emergency communication center

**DOI:** 10.1186/s13049-024-01201-5

**Published:** 2024-04-12

**Authors:** Léo Charrin, Nicolas Romain-Scelle, Christian Di-Filippo, Eric Mercier, Frederic Balen, Karim Tazarourte, Axel Benhamed

**Affiliations:** 1 Service SAMU-Urgences, Centre Hospitalier Universitaire Édouard Herriot, Université Claude Bernard Lyon 1, 5 Place d’Arsonval, 69437 Lyon, France; 2 Department of Biostatistics and Public Health, Hospices Civils de Lyon, Université Claude Bernard Lyon 1, Lyon, France; 3 CHU de Québec-Université Laval Research Centre, Québec, Québec Canada; 4 Emergency Department, University Hospital of Toulouse, 31059 Toulouse, France

**Keywords:** Dyspnea, Prehospital, Emergency communication center, Advanced life support

## Abstract

**Background:**

Shortness of breath is a common complaint among individuals contacting emergency communication center (EMCCs). In some prehospital system, emergency medical services include an advanced life support (ALS)-capable team. Whether such team should be dispatched during the phone call or delayed until the BLS-capable paramedic team reports from the scene is unclear. We aimed to evaluate the impact of delayed MMT dispatch until receiving the paramedic review compared to immediate dispatch at the time of the call on patient outcomes.

**Methods:**

A cross-sectional study conducted in Lyon, France, using data obtained from the departmental EMCC during the period from January to December 2019. We included consecutive calls related to adult patients experiencing acute respiratory distress. Patients from the two groups (immediate mobile medical team (MMT) dispatch or delayed MMT dispatch) were matched on a propensity score, and a conditional weighted logistic regression assessed the adjusted odds ratios (ORs) for each outcome (mortality on days 0, 7 and 30).

**Results:**

A total of 870 calls (median age 72 [57–84], male 466 53.6%) were sought for analysis [614 (70.6%) “immediate MMT dispatch” and 256 (29.4%) “delayed MMT” groups]. The median time before MMT dispatch was 25.1 min longer in the delayed MMT group (30.7 [26.4–36.1] vs. 5.6 [3.9–8.8] min, *p* < 0.001). Patients subjected to a delayed MMT intervention were older (median age 78 [66–87] vs. 69 [53–83], *p* < 0.001) and more frequently highly dependent (16.3% vs. 8.6%, *p* < 0.001). A higher proportion of patients in the delayed MMT group required bag valve mask ventilation (47.3% vs. 39.1%, *p* = 0.03), noninvasive ventilation (24.6% vs. 20.0%, *p* = 0.13), endotracheal intubation (7.0% vs. 4.1%, *p* = 0.07) and catecholamine infusion (3.9% vs. 1.3%, *p* = 0.01). After propensity score matching, mortality at day 0 was higher in the delayed MMT group (9.8% vs. 4.2%, *p* = 0.002). Immediate MMT dispatch at the call was associated with a lower risk of mortality on day 0 (0.60 [0.38;0.82], *p* < 0.001) day 7 (0.50 [0.27;0.72], *p* < 0.001) and day 30 (0.56 [0.35;0.78], *p* < 0.001)

**Conclusions:**

This study suggests that the deployment of an MMT at call in patients in acute respiratory distress may result in decreased short to medium-term mortality compared to a delayed MMT following initial first aid assessment.

**Supplementary Information:**

The online version contains supplementary material available at 10.1186/s13049-024-01201-5.

## Background

Shortness of breath is a common complaint among individuals seeking consultation in emergency departments (EDs) or contacting emergency medical communication center (EMCCs), and is one of the most frequent symptoms of adults transported by ambulance [1–3]. The primary goal of EMCCs is to identify critical situations and prioritize dispatch for prompt assistance. However, the underlying causes of shortness of breath encompass a broad spectrum of conditions, including potentially life-threatening conditions such as hypoxemic and/or hypercapnic respiratory failure, acute heart failure, pulmonary embolism, and drug overdose, contributing to the nonspecific nature of this complaint. Although certain protocols have been developed to enhance the identification of critical situations, recognizing severe cases remains a significant challenge for healthcare professionals [4–6].

Many prehospital emergency medical services (EMS) operate on a two-tiered, involving both basic life support (BLS)-capable and advanced life support (ALS)-capable physician-led teams. In France, nonphysician-trained dispatchers receive the call, gather preliminary information, and aim to identify immediate life-threatening cases before transferring the call to the attending physician. In situations where no life-threatening situations are identified, a fire brigade (FB) unit or a paramedic ambulance, both BLS-trained may be dispatched to the scene to provide BLS and transport the patient to a healthcare facility. They may provide oxygen via canula or mask but are not trained to any advanced techniques (including supraglottic devices) and cannot administer any oral or intravenous medication [7, 8]. In the most severe cases, a mobile medical team (MMT) can be dispatched to implement ALS techniques, including mechanical ventilation. A MMT can be dispatch during the phone call if a critical situation is identified, or it may be delayed until the BLS-capable paramedic reports from the scene if the patient condition is more severe than expected at the initial call.

Although the benefit of the “stay and play” strategy vs. “scoop and run” is still debated, reducing the time interval before initiating certain ALS procedures holds paramount importance for time-sensitive conditions [9, 10]. For instance, prehospital ALS interventions have shown an increased likelihood of 1-month survival in traumatic out-of-hospital cardiac arrest [11], while prehospital noninvasive ventilation has been found to reduce the need for in-hospital endotracheal intubation in respiratory failure [12, 13]. Nevertheless, the impact of the time interval before dispatching an ALS-capable team to manage patients presenting with respiratory distress in the prehospital setting remains unexplored. Therefore, our study aimed to evaluate the effect of delayed MMT dispatch (i.e. until receiving the paramedic review) compared to immediate MTT dispatch (i.e. at the time of the initial call to EMCC) on patient mortality.

## Methods

### Study design and setting

We conducted a cross-sectional study in Lyon, France, using data obtained from the departmental EMCC during the period from January 1, 2019, to December 31, 2019. The corresponding geographical area has a population of 1.9 million inhabitants, and an average of 700,000 calls are managed annually.

Prehospital EMS in France operate on a 24-hour physician-led system. Access to the EMCC is available nationwide through a single free national telephone number “15”. Initial call receptions are handled by nonphysician professionals known as assistants, whose purpose is to promptly identify immediate life-threatening cases and collect essential information, including patient identity and location. Subsequently, calls are transferred to one attending physician, who may be a general practitioner or an emergency physician, depending on the initial severity assessment. In comparison to other nations where dispatcher decisions are guided by standardized protocols like the “Medical Priority Dispatch System”, in French EMCC, the decision-making process relies primarily on the judgment of physicians.

**Fig. 1:**
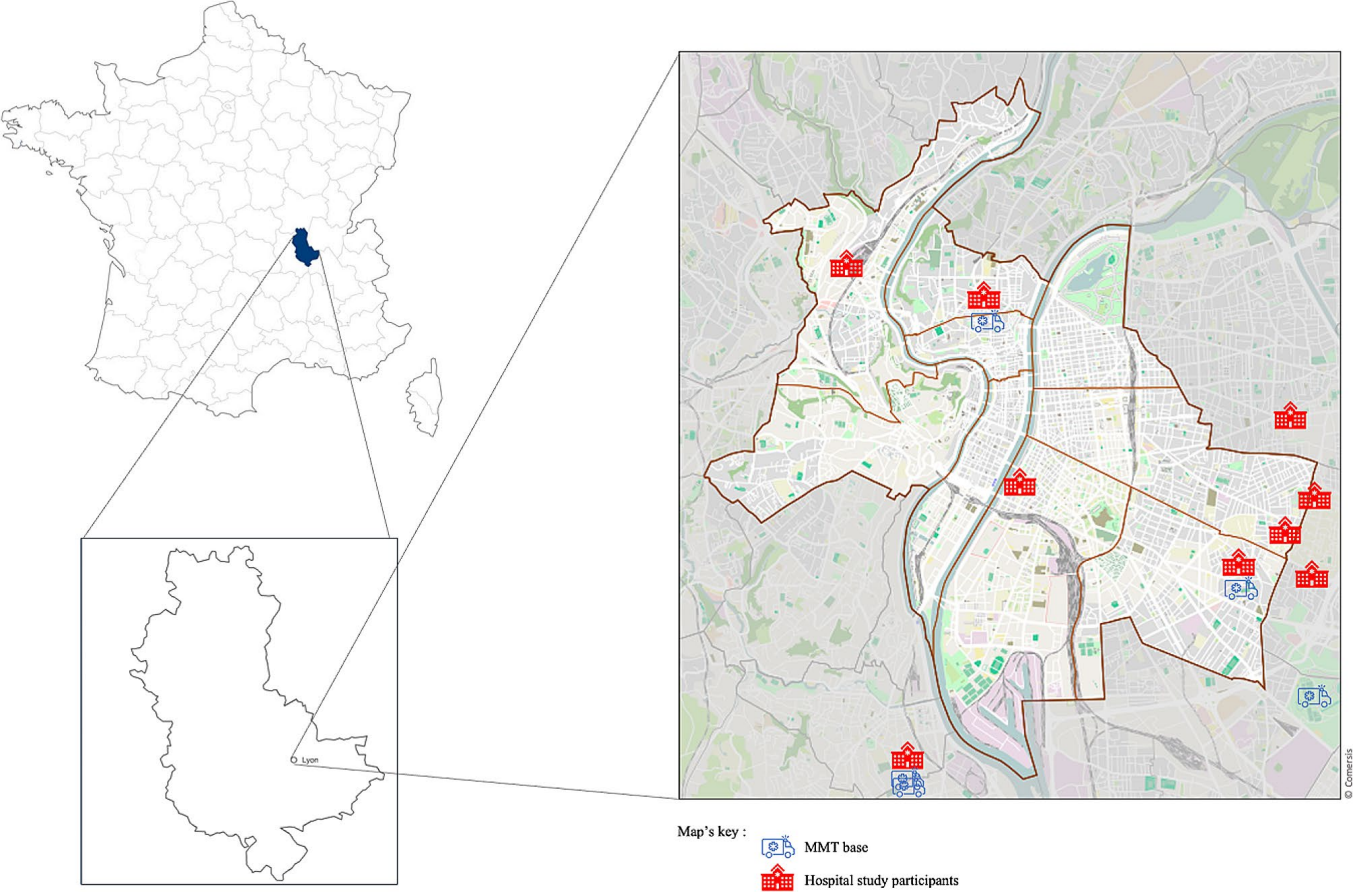
Map showing the distribution of MMTs and study partner hospitals

In cases where no critical situations are identified, a paramedic ambulance may be dispatched to the scene to implement basic life support (BLS). This includes administering oxygen through a cannula or mask but does not extend to advanced procedures or the use of medication, even if a critical condition such as cardiac arrest unexpectedly arises. In such instances, the paramedics will relay a situation report to the EMCC to seek further support. In contrast, both a paramedic ambulance and a MMT are systematically dispatched to the scene for suspected life-threatening cases. The MMT consists of an emergency physician or an anesthesiologist-intensivist physician, a nurse, an ambulance driver, and a medical resident in academic centers. MMTs can be transported by ground ambulance or helicopter and are strategically distributed throughout the country at hospital-based locations (Fig. 1).

Following a basic clinical evaluation, both FB and paramedics are required to contact the dispatching physician for further decision-making. They determine whether the patient is stable and suitable for transportation or if additional assistance is needed. In cases requiring additional assistance, a MMT may be dispatched later (delayed MMT group). Similarly, in cases where immediate MMT dispatch is warranted, the on-scene physician and the dispatching physician collaborate to determine the most appropriate healthcare facility for patient(s) referral.

### Population

We included consecutive calls related to patients aged 18 years or older who were labeled as experiencing “acute respiratory distress” and recorded as such in the electronic medical chart record system. We excluded calls that were not handled by a physician dispatcher and those that did not result in the dispatch of an MMT. Additionally, patients who did not require or refuse transportation, those admitted to private centers (because of limited data accessibility), and those identified in cardiac arrest upon the first healthcare provider arrival were also excluded from the analysis. Patients with missing data related to a variable of the propensity score, or outcome were also excluded. Finally, MMT interventions conducted within healthcare facilities were excluded from the analysis due to the potential confounding effects of preliminary treatments initiated prior to MMT arrival, which could impact patient outcomes. The included patients were subsequently divided into two groups based on whether they received immediate MMT dispatch at the time of the initial emergency call or delayed MMT dispatch after a paramedic team evaluation and review.

### Outcomes

The primary outcome was mortality on day 0, while the secondary outcomes were mortality on day 7 and 30.

### Variables

Patient demographics, comorbidities, prehospital clinical findings, timings, prehospital management, and patient outcomes were extracted from patient electronic medical charts by a post-graduate year 3 emergency medicine resident (LC). Comorbidities were divided into four categories: cardiovascular (chronic high blood pressure, chronic heart failure, ischemic cardiomyopathy, arrhythmia), neurological (past history of stroke), pulmonary diseases (chronic obstructive pulmonary disease, asthma, pulmonary embolism, pulmonary fibrosis) and active cancer. Patients level of dependency was also collected based on the AGGIR (*Autonomie Gérontologie Groupes Iso Ressources*) scale from 1 to 6 [14, 15]. High-level of dependency patients were defined as GIR 1 and 2 patients. GIR 1 pertains to individuals confined to bed or chair, with severely impaired cognitive functions, requiring the continuous presence of a caregiver or end-of-life individuals. GIR 2 corresponds individuals confined to bed or chair, whose cognitive functions are not entirely impaired, and whose condition demands assistance for most daily activities or individuals with impaired cognitive functions, but who can move around and require constant monitoring. Vital status was obtained from the French death registry.

### Statistical analysis

#### Description and comparison of the two populations

Quantitative variables were expressed by their medians and interquartile ranges (IQR). Qualitative variables were expressed by their frequencies and percentages. Patient characteristics were compared between the two populations (immediate MMT and delayed MMT) using the Wilcoxon rank sum test for quantitative variables, while Pearson’s chi-squared test or Fisher’s exact test was used for qualitative variables. Time to event data was presented using Kaplan-Meier survival curves.

### Calculation of the propensity score and the matching method

Patients from the two groups were matched on a propensity score to mitigate bias caused by confounders. A logistic regression model with all second-order interactions was used to estimate the propensity score. A total of 7 covariates were selected a priori according to their clinical relevance: patient’s age, permanent residency in care home, comorbidities: cardiovascular disease, pulmonary embolism, chronic obstructive pulmonary disease, asthma, and distance between the patient location and the MMT base. For the propensity score, the dependent variable was the delay before MMT dispatch defined as delayed or immediate MMT dispatch. A 1:1 optimal propensity score matching without replacement was conducted.

Bias reduction through propensity score matching was assessed by calculating standardized absolute mean differences (SADs) in each baseline characteristic between the two populations. An SAD of less than 0.1 was considered acceptable to indicate a negligible difference between the two populations.

### Evaluation of outcomes

For each outcome (mortality on day 0, 7 and 30), we estimated the effect size of immediate MMT (compared to delayed MMT) using parametric g-formula [16]on the matched sample. For each outcome, a logistic regression model was fitted on the matched dataset, adjusted for the covariates used to conduct the matching procedure. Then, we predicted the counterfactual outcomes for each subject (two predictions per individual: with delayed MMT and immediate MMT). The final estimate was the mean of individual level effect sizes. Standard errors were computed using the delta method. All analysis were conducted in R 4.1.0. Matching was conducted using the MatchIt package [17]with “optimal [18, 19]” setting, parametric g-formula computation was conducted using the marginal effects package.

### Sensisitivy analyses

Mortality at day 0 was evaluated across various sub-populations of the study to assess the sensitivity of the results to deviations from positivity (patients for whom it is highly unlikely that a dispatch occurred at the call), which could lead to a biased estimation of the estimated effect.

The selection criteria (age > 85, high level of dependency [GIR 1 or 2 patients]) were applied before the matching procedure. Relative risks were estimated using the same procedure as described for the main analysis.

## Results

### Patient characteristics

**Fig. 2:**
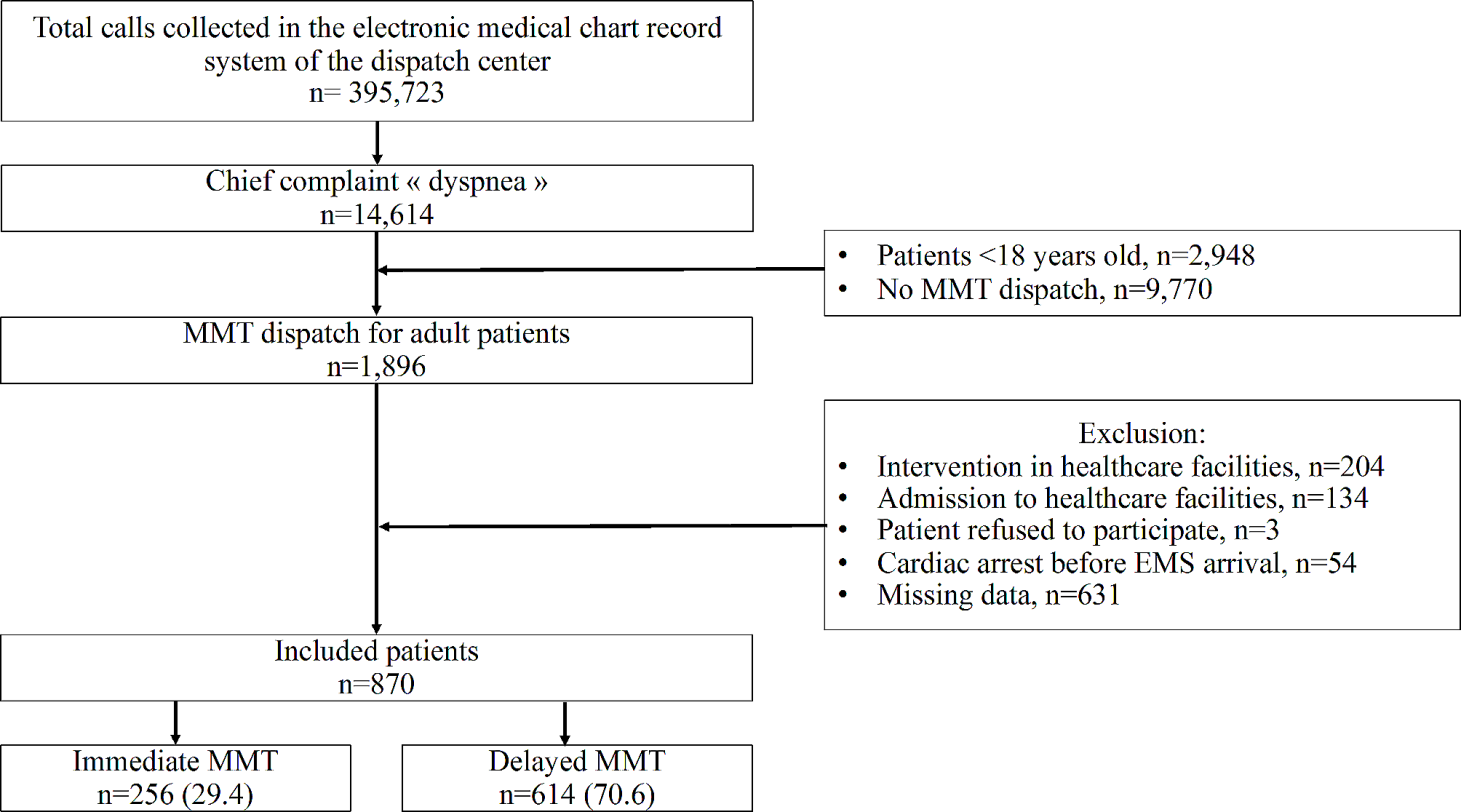
Flow chart of the study MMT: Mobile medical team, EMS: Emergency medical services

During the study period, a total of 14,616 calls were received by the EMCC, resulting in the dispatch of an MMT in 1,896 adult cases. From this subset, 870 calls (median age 72 [57–84], male 466 53.6%) were sought for analysis (Figs. 2) and 614 (70.6%) cases were classified as immediate MMT dispatch, while the remaining 256 (29.4%) belonged to the delayed MMT group. The median time before MMT dispatch was 25.1 min longer in the delayed MMT group (30.7 [26.4–36.1] vs. 5.6 [3.9–8.8] min, *p* < 0.001).

Compared to patients in the immediate MMT group, those subjected to a delayed MMT intervention were older (median age 78 [66–87] vs. 69 [53–83], *p* < 0.001) and more frequently highly dependent (16.3% vs. 8.6%, *p* < 0.001). There were no significant differences in terms of sex between the two groups (male 52.7% vs. 53.9%, *p* = 0.75). Patients in the delayed MMT group exhibited lower systolic blood pressure (134 [110–163] vs. 141 [120–167] mmHg, *p* = 0.002) and lower oxygen saturation levels (86 [75–95] vs. 92 [80–98] %, *p* < 0.001, Table 1).

### Prehospital MMT management and diagnoses

Table 2 presents the prehospital management and interventions provided by MMTs at the scene. A significantly higher proportion of patients in the delayed MMT group required respiratory support: bag valve mask ventilation (47.3% vs. 39.1%, *p* = 0.03), noninvasive ventilation (24.6% vs. 20.0%, *p* = 0.13) or endotracheal intubation (7.0% vs. 4.1%, *p* = 0.07). A larger proportion of patients in the delayed MMT group also required catecholamine infusion (3.9% vs. 1.3%, *p* = 0.01). Conversely, patients in the delayed MMT group received a corticosteroid bolus (5.5% vs. 15.3%, *p* < 0.001), a dexchlorpheniramine bolus (1.2% vs. 7.5%, *p* < 0.001), and an intramuscular injection of epinephrine (0% vs. 2.8%, *p* = 0.005) less frequently.

Notably, a majority of patients from both groups were referred to an ED (56.7% vs. 59.6% in the immediate and delayed MMT groups, respectively).

The most frequent final diagnoses in the immediate MMT group were cardiogenic pulmonary edema (18.6%), exacerbation of chronic obstructive pulmonary disease (COPD, 13.8%), and anaphylaxis (11.2%), while in the delayed MMT group, the most frequent diagnoses were exacerbation of COPD (18.8%), cardiogenic pulmonary edema (16.4%), and hypoxic pneumonia (10.5%, Table 3).

### Patient outcomes and impact of delayed MMT dispatch

**Fig. 3:**
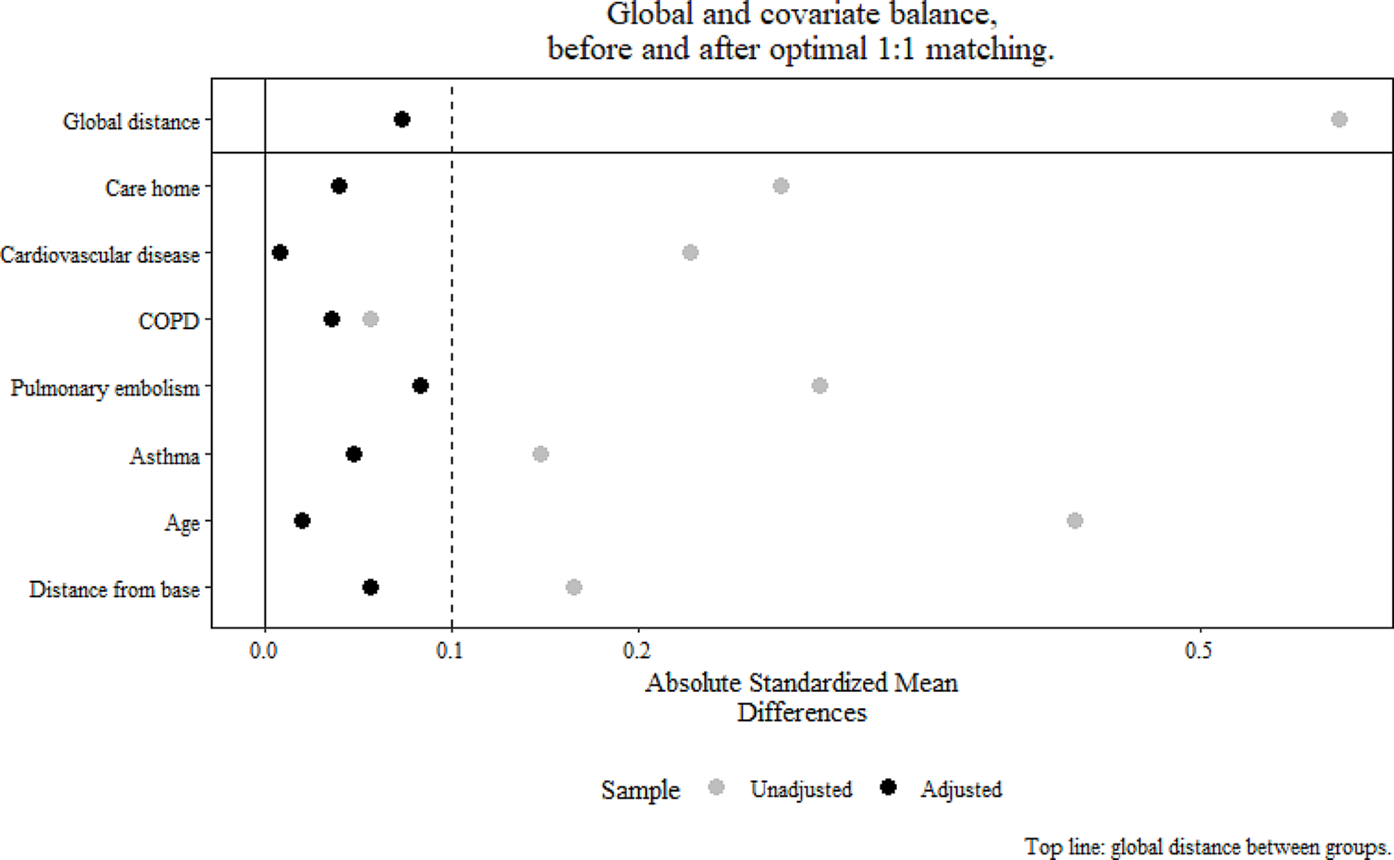
Absolute standardized mean differences COPD: chronic obstructive pulmonary disease

After propensity score matching, absolute standardized mean differences below 10% were achieved for every variable, thereby confirming the validity of the matching procedure (Fig. 3). Propensity score matching generated 256 patient pairs.

**Fig. 4:**
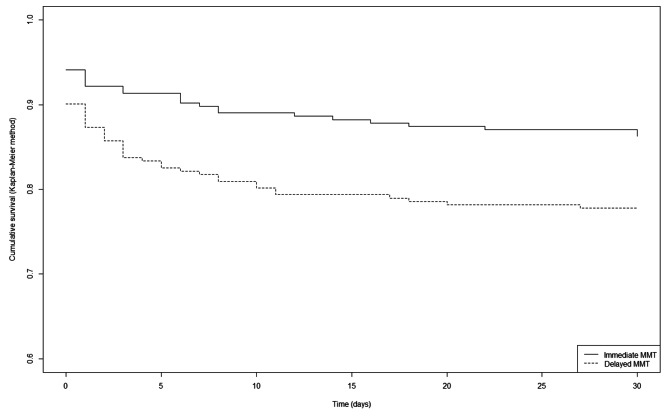
Survival between immediate and delayed mobile medical team dispatch groups

Mortality at day 0, 7 and 30 were significantly higher in the delayed MMT group (9.8% vs. 4.2%, *p* = 0.002, 18% vs. 7.2%, *p* < 0.001 and 23.4% vs. 10.7%, *p* < 0.001, respectively). Immediate MMT dispatch at the call was independently associated with a lower risk of mortality on day 0 (RR 0.60 [0.38;0.82], *p* < 0.001), on day 7 (RR 0.50 [0.27;0.72], *p* < 0.001) and on day 30 (RR 0.56 [0.35;0.78], *p* < 0.001), as shown in the Kaplan‒Meier curves (Fig. 4).

Sensitivity analyses found consistent results regarding mortality on day 0 when excluding specific demographic groups, including patients aged ≥ 85 years (RR 0.36 [0.14;0.58], *p* = 0.002), GIR 1 patients (RR 0.35 [0.33;0.76], *p* < 0.001), and a combined group of patients aged ≥ 85 years or classified as GIR 1 (RR 0.37 [0.16;0.58], *p* < 0.001).

**Table 1:** Patient characteristics and timings

	Total population *n* = 870	Immediate MMT *n* = 614 (70.6)	Delayed MMT *n* = 256 (29.4)	*p* value	Missing values
Age, years	72 [57–84]	69 [53–83]	78 [66–87]	**< 0.001**	0
Sex, male	466 (53.6)	331 (53.9)	135 (52.7)	0.75	0
Comorbidities					
Cardiovascular	536 (61.6)	358 (58.3)	178 (69.5)	**0.002**	0
Neurological	196 (22.5)	130 (21.2)	66 (25.8)	0.14	0
Respiratory	332 (38.2)	229 (37.3)	103 (40.2)	0.42	0
Cancer	167 (19.2)	114 (18.6)	53 (20.7)	0.51	0
Living in a care home	106 (12.2)	60 (9.8)	46 (18.0)	**< 0.001**	0
Active smoker	155 (17.8)	116 (18.9)	39 (15.2)	0.21	0
High level of dependency	91 (11.0)	50 (8.6)	41 (16.3)	**< 0.001**	39 (4.5)
Caller status, health professional	190 (21.9)	125 (20.5)	65 (25.4)	0.11	4 (0.5)
Time from call to BLS-team dispatch^a^, seconds	120 [45–247]	110 [25–215]	153 [71–307]	**< 0.001**	0
Type of MMT, helicopter	9 (1.0)	5 (0.8)	4 (1.6)	0.68	0
Time from call to MMT dispatch, min	7.9 [4.5–25.2]	5.6 [3.9–8.8]	30.7 [26.4–36.1]	**< 0.001**	0
Time from call to MMT arrival at the scene, min	81.0 [65.4–104.0]	72.5 [61.1–90.6]	103.7 [88.4-122.3]	**< 0.001**	243
Time from MMT dispatch to arrival at the scene, min	8.7 [5.8–11.6]	8.3 [5.5–11.2]	9.4 [6.8–13.0]	**0.008**	317 (36.4)
Time from MMT dispatch to the end of the intervention, min	89 [69–110]	87 [68–109]	91 [76–115]	**0.014**	36 (4.1)
MMT clinical evaluation					
Systolic blood pressure, mmHg	140 [120–166]	141 [120–167]	134 [110–163]	**0.002**	23 (2.6)
Heat rate, beats/min	101 [85–120]	100 [84–120]	103 [86–123]	0.23	37 (4.2)
O_2_ saturation, %	90 [78–97]	92 [80–98]	86 [75–95]	**< 0.001**	25 (2.8)
Respiratory rate	32 [25–40]	32 [25–40]	32 [25–40]	0.8	435 (50)
Temperature, Celsius degree	36.8 [36.1–37.3]	36.7 [36.1–37.2]	36.8 [36.1–37.6]	0.22	386 (44.3)
Glasgow coma scale	15 [14–15]	15 [15–15]	15 [14–15]	**0.006**	41 (4.7)
Diaphoresis	161 (27.3)	119 (28.3)	42 (25.0)	0.42	281 (32.3)
Signs of respiratory distress^b^	351 (48.8)	240 (47.2)	111 (52.4)	0.21	150 (17.2)
Cyanosis	43 (4.9)	34 (5.5)	9 (3.5)	0.21	0
Mottled Skin	51 (5.9)	35 (5.7)	16 (6.2)	0.75	0
Unable to complete sentences	127 (28.2)	94 (29.5)	33 (25.0)	0.36	419 (48.2)
Chest pain	125 (18.8)	89 (19.1)	36 (18.1)	0.77	207 (23.8)

^a^Whether the call was from patient him/herself, family, or a tierce person

^b^Includes retraction, thoracoabdominal asynchrony, restless

**Table 2:** Prehospital patient care

	Total population *n* = 870	Immediate MMT *n* = 614 (70.6)	Delayed MMT *n* = 256 (29.4)	*p* value
Oxygen supply				
Nasal canula	17 (2.0)	10 (1.6)	7 (2.7)	0.28
Bag valve mask	361 (41.5)	240 (39.1)	121 (47.3)	**0.03**
Noninvasive ventilation	186 (21.4)	123 (20.0)	63 (24.6)	0.13
Endotracheal intubation	43 (4.9)	25 (4.1)	18 (7.0)	0.07
Catecholamine IV, norepinephrine	18 (2.1)	8 (1.3)	10 (3.9)	**0.01**
Diuretic IV, furosemide	107 (12.2)	67 (10.9)	40 (15.6)	0.07
Vasodilatator IV, isosorbide dinitrate	117 (13.4)	81 (13.2)	36 (14.1)	0.74
Corticosteriod bolus	108 (12.4)	94 (15.3)	14 (5.5)	**< 0.001**
Antihistamine IV, dexchlorpheniramine	49 (5.6)	46 (7.5)	3 (1.2)	**< 0.001**
Epinephrine, IM injection	17 (2.0)	17 (2.8)	0 (0)	**0.005**
Heparin, IV	19 (2.2)	11 (1.8)	8 (3.2)	0.21
Aspirin, IV	49 (5.6)	36 (5.9)	13 (5.1)	0.65
Nitroglycerin, sublingual spray	28 (3.2)	23 (3.7)	5 (2.0)	0.17
In-hospital referral				0.90
Emergency department	511 (58.8)	366 (59.6)	145 (56.7)	
Intensive care unit	290 (33.3)	196 (31.9)	94 (36.7)	
Catheterization laboratory	19 (2.2)	14 (2.3)	5 (2.0)	
Other	4 (0.5)	4 (0.7)	0 (0)	
No hospital admission	46 (5.3)	34 (5.5)	12 (4.7)	
In-hospital admission after ED	369 (42.4)	246 (40.1)	123 (48.0)	**< 0.001**
ICU admission after ED	47 (9.2)	39 (10.7)	8 (3.1)	0.07

No missing value

**Table 3:** Final diagnoses and patient outcomes

	Total population *n* = 870	Immediate MMT *n* = 614 (70.6)	Delayed MMT *n* = 256 (29.4)	*p* value
Pulmonary	300 (34.5)	196 (31.9)	104 (40.6)	**0.02**
COPD exacerbation	133 (15.3)	85 (13.8)	48 (18.8)	
Hypoxic pneumonia	59 (6.8)	32 (5.2)	27 (10.5)	
Asthma exacerbation	58 (6.6)	53 (8.6)	5 (2.0)	
Other	37 (4.3)	17 (2.8)	20 (7.9)	
Pulmonary embolism	9 (1.0)	5 (0.8)	4 (1.6)	
Pleural effusion	3 (0.3)	3 (0.5)	0 (0)	
Pneumothorax	1 (0.1)	1 (0.2)	0 (0)	
Cardiovascular	261 [30]	181 (29.5)	80 (31.3)	0.63
Cardiogenic pulmonary edema	156 (17.9)	114 (18.6)	42 (16.4)	
Global acute heart failure	23 (2.6)	10 (1.6)	13 (5.1)	
STEMI	21 (2.4)	15 (2.4)	6 (2.3)	
Chest pain	19 (2.3)	15 (2.5)	4 (1.6)	
Arrhythmia	16 (1.8)	10 (1.6)	6 (2.4)	
NSTEMI	12 (1.4)	9 (1.5)	3 (1.2)	
High-grade AV block	7 (0.8)	5 (0.8)	2 (0.8)	
Cardiogenic shock	5 (0.6)	2 (0.3)	3 (1.2)	
Medical cardiac arrest	2 (0.2)	1 (0.2)	1 (0.4)	
Other	151 (17.4)	100 (16.3)	51 (19.9)	0.21
Anaphylaxis	74 (8.5)	69 (11.2)	5 (2.0)	**< 0.001**
Psychiatric^a^	36 (4.1)	29 (4.7)	7 (2.7)	0.10
Ear-nose-throat^b^	26 (3.0)	20 (3.3)	6 (2.3)	0.66
Neuro^c^	22 (2.5)	19 (3.1)	3 (1.2)	0.15
Mortality				
Day 0	51 (5.9)	26 (4.2)	25 (9.8)	**0.002**
Day 7	90 (10.3)	44 (7.2)	46 [18]	**< 0.001**
Day 30	126 (14.5)	66 (10.7)	60 (23.4)	**< 0.001**

No missing value.

AV: atrioventricular, COPD: chronic obstructive pulmonary disease, NSTEMI: non-ST elevation myocardial infarction, STEMI: ST elevation myocardial infarction.

^a^Includes panic attack, anxiety, somatization, self-poisoning.

^b^Includes: tracheo-bronchial foreign body, epiglottitis, hemoptysis, laryngitis, tonsillitis.

^c^Includes: stroke, seizure, status epilepticus

## Discussion

The present study yields findings suggesting that immediate dispatch of MMT for patients experiencing acute respiratory failure is associated with a decreased risk of mortality compared to delayed MMT dispatch until receiving the paramedics review.

Similar to the management of severe trauma cases, acute respiratory failure is a time-dependent critical situation that may benefit from prompt interventions by ALS-capable teams as soon as the prehospital setting. Elsewhere Stiell et al. evaluated the impact of a program to provide ALS for patients with out-of-hospital respiratory distress. They found that the rate of death decreased significantly, from 14.3 to 12.4%, from the BLS phase to the ALS-support phase (aOR, 1.3; 95% CI, 1.1 to 1.5) [20]. However, the impact of delayed dispatch of an ALS-capable team in the prehospital setting had never been evaluated before. Herein, one hypothesis is that a shorter delay until MMT arrival may have allowed faster initiation of certain therapies (e.g., noninvasive ventilation, beta-agonist), which contributed to mitigating the number of patients requiring more invasive care (e.g., orotracheal intubation), which has been reported to be associated with adverse effects, including mortality [21, 22]. In line, the need for endotracheal intubation was twice as high in the delayed MMT group compared to the immediate MMT group (7.0% vs. 4.1%). Considering the very limited prerogatives of BLS units within the French prehospital system, the delay in dispatching MMTs could be more critical compared to some other systems where paramedics may be trained to use supraglottic airway and administer b2-agonist, epinephrine, corticosteroids and/or positive end-expiratory pressure for instance.

In the present study, patients in the delayed MMT group presented with a lower oxygen saturation (median 86% vs. 92%), a lower SBP (median 134 mmHg vs. 141 mmHg), and more frequent signs of respiratory distress (52.4% vs. 47.2%). Another hypothesis is therefore that patients’ conditions in the delayed MMT group may have worsened since their call to the EMCC. The difference in terms of mortality between the two groups could also be influenced by decision of withholding and/or withdrawing some treatments. It also cannot be excluded that our finding relies on heterogeneous dispatcher performance to recognize those signs across groups. Finally, while immediate dispatch relies on incomplete information and may lead to overtriage, delayed dispatch incorporates scene-derived data, allowing for a more thorough assessment and a higher likelihood of identifying individuals with higher acuity conditions. Interestingly, a recent study has found that the identification of certain factors during a patient’s initial call is associated with an early need for respiratory support. These factors include the use of chronic β2-mimetics medication, altered speech ability, cyanosis, sweating, and altered consciousness [23]. Recognizing these factors may prompt the timely dispatch of a MMT for immediate intervention. It is also noteworthy that the final diagnoses were not equally distributed between groups.

As suggested before, it is plausible that the immediate interventions offered by MMTs effectively address the critical phase in certain specific cases, resulting in reduced prehospital mortality. However, the sustained impact of immediate MMT dispatch on day 30 is questionable and it cannot be ruled out that our findings are subject to unmeasured potential confounding interventions unrelated to MMT intervention. Indeed, as patients progress through subsequent phases of care, such as hospitalization and specialized treatments, the discernible influence of immediate MMT dispatch may diminish due to the complex nature of patient management and the involvement of various healthcare providers. Therefore, the sustained impact of immediate MMT dispatch on day 30 will need to be further evaluated in future studies.

It is also worth noting that the population with delayed MMT dispatch was significantly older (median age + 9 years), which raises concerns about the effectiveness of the current emergency medical dispatch response for older adults in EMCCs. Similarly, previous studies have reported that this specific population was less likely to be transported to a high level trauma care trauma center than their younger counterpart [24, 25].

From a clinical point of view, the results of the present study suggest that immediate MMT dispatch may have a positive impact on patient outcomes. However, it is important to consider that MMTs represent a limited and expensive medical resource. Further research is therefore still warranted to better identify, at the initial call, the patient characteristics and complaints that should trigger an immediate MMT dispatch [26, 27]. One potential avenue may involve video communication integration, providing a more comprehensive assessment of patient conditions and facilitating the dispatch process [28, 29]. Its benefit has already been demonstrated in patient assessment but also in guiding CPR maneuvers [30]. Verbatim analysis may also be an avenue for future research, as has already been demonstrated that in patients with suspected cardiorespiratory arrest [31].

### Strengths and limitations

Some limitations need to be acknowledged in this study. First, the retrospective design introduces inherent limitations. A randomized clinical trial would have been ideal for determining the true impact of immediate MMT dispatch. However, conducting such a trial would have presented major ethical challenges. Nonetheless, the use of a propensity score represents the most suitable approach to isolate and evaluate the impact of timing before MMT dispatch. The inclusion of institutionalization status in the propensity score analysis allowed for the consideration of frailty, which is not routinely assessed by the dispatchers by a score, while it may have impacted their decision. Second, we could not account for decisions regarding withholding or withdrawing life-sustaining treatments. These decisions can significantly influence patient outcomes and may have affected the results observed. Third, the exact reasons behind the differences in MMT dispatch timing between patients were unknown. It is plausible that the severity was underestimated during the initial call or that the patient condition worsened over time. However, it is not excluded that in some cases, immediate MMT was not feasible due to the unavailability of MMTs or due to concerns about the distance between the MMT base and the patient’s location. Dispatchers might have opted for an evaluation by an BLS-capable paramedic team to better assess the situation.

## Conclusion

This study suggests that the deployment of an MMT at call in patients in acute respiratory distress may result in decreased short to medium-term mortality compared to a delayed MMT following initial first aid assessment. Enhancing the initial triage process is essential to ensure timely and appropriate MMT deployment to patients with symptoms of acute respiratory distress.

### Electronic supplementary material

Below is the link to the electronic supplementary material.

Supplementary Material 1

## Data Availability

The datasets used and/or analysed during the current study are available from the corresponding author on reasonable request.

## Abbreviations

ALS: Advance Life Support
AGGIR: *Autonomie Gérontologie Groupes Iso Ressources*
BLS: Basic Life Support
COPD: Chronic Obstructive Pulmonary Disease
CPR: Cardiopulmonary Resuscitation
ED: Emergency Department
EMCCs: Emergency Medical Communication Center
EMS: Emergency medical services
FB: Fire Brigade
GIR: *Groupe Iso-Ressource*
IQR: Interquartile Ranges
MMT: Mobile Medical Team
SAD: Standardized Absolute Mean Differences
