# Supplementary material for: Impact of delayed mobile medical team dispatch for respiratory distress calls: a propensity score matched study from a French emergency communication center
Source: Scand J Trauma Resusc Emerg Med. 2024 Apr 12;32:27. doi: 10.1186/s13049-024-01201-5 (PMC11010329; doi:10.1186/s13049-024-01201-5)
Supplement: Supplementary file 1 — Supplementary Material 1 [file 13049_2024_1201_MOESM1_ESM.docx]

We first would like to thank very much the reviewers for their positive comments, thorough review and valuable suggestions, which have significantly improved our manuscript. Their expertise and insights are greatly appreciated and have been instrumental in enhancing the quality of our work.

**Reviewer #1:** Reviewer: Dr. Johannes von Vopelius-Feldt

1. Statistical analysis: 'The dependent variable was the delay before MMT dispatch'. Can the authors clarify that this was for the propensity score calculation (not the final analysis). Also, do the authors mean the delayed dispatch or the actual minutes in delay? Just needs clear language to avoid confusion.

Authors response: Thank you for this comment, we agree that this point should be clarified, it has been done accordingly.

2. Outcomes and Evaluations of outcomes sections. These are missing the 30 day mortality outcome (which should be the primary outcome as it's most patient centred).

Authors response: Thank you for your vigilance, 30-day mortality has been added in this paragraph.

3. Results. The text states that all 'absolute standardized mean differences below 10% were achieved for every variable'. However, table 3 shows that some reasonably big differences remain between groups. There are higher rates of COPD and hypoxic pneumonia in the delayed group, both have frequently poor prognosis. On the other hand, anaphylaxis and asthma are much more common in the immediate dispatch group and, if patients don't arrest early, prognosis is usually excellent. This remaining imbalance would certainly affect the measured survival rates. My understanding is that, if imbalance remains after PSM, a conditional logistic regression should include the variables which are poorly balanced, to further adjust for this.

Authors response: Table 3 focuses on the final diagnosis after hospital admission in the entire cohort. As such those results cannot be considered in defining the variable set for the propensity score matching procedure. Nevertheless, as depicted in Figure 3, after propensity score matching, the attainment of absolute standardized mean differences below the threshold of 10% is evidenced for the other variables including major comorbidities (COPD and asthma) metrics. Following common practices in PSM, all the covariates used for the estimation of the propensity score are included in the primary outcome model.

4. Discussion. The authors hypothesize that patients in the delayed MMT group are sicker and require more intubations due to deterioration during the wait for the MMT. This hypothesis, while plausible, cannot be proven with the data provided and need to be clearly caveated accordingly. In addition, there is an equally or even more plausible, and highly relevant explanation. Immediate dispatch depends on incomplete information and there is a degree of overtriage. Secondary delayed dispatch with information from scene will be more targeted with less overtriage, and therefore identify an on average sicker patient population.

Authors response: Thank you for your insightful comment. we concur with reviewer 1 assessment and appreciate the consideration of alternative explanations provided. The discussion has been enriched accordingly.

5. Discussion 'Specifically, a larger proportion of diseases (e.g., COPD exacerbations 18.8% vs. 13.8%, hypoxemic pneumonia 10.5% vs. 5.2%, acute heart failure 5.1% vs. 1.6%, cardiogenic shock 1.2% vs. 0.3%) associated with a significant risk of early mortality was observed in the delayed MMT group. In contrast, threefold of patients in the immediate MMT group were admitted to the ICU after the ED visit, suggesting that they may be more severe. We therefore do not believe that the difference in terms of diagnosis may explain our findings.' I don't follow this argument. The admission rates to ICU seem pretty balanced between the two groups. And as I outlined in points 3. and 4., I do think they are likely to have an impact on the findings.

Authors response: This section has been removed from the discussion.

6. Do the authors have numbers on how many respiratory dispatch calls currently do not result in MMT dispatch? What would be the impact on call volumes if switching to immediate dispatch for all of these.

Authors response: During the study period, a total of 14,614 calls were related to respiratory conditions. Of these, 2,948 involved patients under the age of 18, and 9,770 did not involve a MMT dispatch at any point during prehospital management. These details have been illustrated in the flowchart (fig 2).

7. Overall, I am very skeptical that a 25min difference in dispatch time for an MMT can have such a dramatic difference in outcomes, particularly one that increases in effect size from 0 to 7 to 30 days. Usually with advanced teams like this, the opposite is the case. Early mortality is more impacted, and then the effect reduces over time. I am fairly certain that the effect observed in this research is heavily influenced by indication bias (delayed MMT dispatch to sicker patients) and that the PSM didn't fully correct for this (due to diagnoses remaining unbalanced). This unfortunately makes it very hard to say with confidence what the actual impact of immediate vs delayed MMT is.

Authors response: Median time from call to MMT arrival at the scene is 30min longer in the delayed group, this can have made a big difference in patient prognosis for the sicker ones with very serious conditions. Indeed in the French prehospital EMS, paramedics can allow administer oxygen but are not trained to any advanced procedures (this includes supraglottic device) or the use of medication, even if a critical condition such as cardiac arrest unexpectedly arises. This has been further detailed in the methods section.

As for long-term impact, we have acknowledged in the discussion section that the sustained impact of immediate MMT dispatch on day 30 is questionable and it cannot be ruled out that our findings are subject to unmeasured potential confounding interventions unrelated to MMT intervention. We also acknowledge that a RCT would have been ideal for determining the true impact of immediate MMT dispatch, however conducting such a trial would present major ethical challenges Nonetheless, the use of a propensity score represents the most rigorous approach to isolate and evaluate the impact of timing before MMT dispatch as it took into account covariates that may influence patient age, frailty and comorbidities. Finally, we noted that a total of 38.3% (n=196+39) patients in the direct MMT group were eventually admitted to the ICU while it was of 39.8% (n=94+8) in the delayed MMT group. Therefore, it is reasonable to assume that the patients were overall equally severely sick in one group as in the other.

The present results should therefore be considered as exploratory and lead to future prospective studies to better understand the ideal dispatch time of MMT for patients with severe respiratory conditions. This has been emphasized in line with reviewer 1 comment.

**Reviewer #2:**

Background: you need to describe/ explain what you mean by paramedic and BLS-unit. Across Europe the paramedic title is not consistent. Some countries consider paramedics as an ALS-provider (e.g. UK, Denmark, Norway).

So I would like to read, what a paramedic is in France / your context. My understanding of the level of your paramedics are what I would consider to be an EMT (emergency medical technician). But please ellaborate this, so the reader knows that you mean and define a paramedic by. What paramedics in France can / cannot do regarded to patient care and therapies. In the UK, Ireland you would be treated by a paramedic and an EMT, not a MD prior to arrival at the A&E. in Norway and Denmark you might be seen by an anaestesiologist in the prehospital phase, but not necessarily due to most cases are treated by the ambulance personnel.

Authors response: Thank you for this comment, we agree that this point should be clarified, it has been done accordingly. Paramedic in France cannot provide any medication but oxygen and that excludes advanced airway management.

Page 4, line 76: there is a minor fault in the word paramédic. There should be an é, but an e.

Authors response: Thank you for your vigilance, this has been corrected accordingly.

In the Method-section: once again it is not clear whether it is a paramedic or an EMT on the BLS-unit (it should be clear what to expect from a paramedic / EMT in your context).

Authors response: This has been clarified in line with reviewer 2 comment.

Results: You mention 2,076 cases but "only" analyse 870. Where did the others go? - they are not argued for in the text nor in your figure 2 (flow-chart).

Authors response: We have re-evaluated this number and identified a mistake in how the results were initially presented. Accordingly, it has been adjusted to 1,896, representing a total of 14,614 calls. A total of 2,948 cases involving children and 9,770 instances where no mobile medical team was dispatched where excluding leading to a total of 1,896 cases. We then applied exclusion criteria. The flowchart presented in Figure 2 has been updated to improve understanding of results.

Discussion: Can you ellaborate more regarding the use / less use of IM Epinephrine versus IV Catecholamines? Why are there so few anaphylaxis-cases in your "delayed MMT group"? Does the dispatcher give anaphylaxis a different response type due to other reasons than "dyspnea" / "shortness of breath"?

Authors response: The higher incidence of intramuscular epinephrine (IM Epi) administration in the MMT group is likely attributable to the higher occurrence of anaphylaxis within this group. The reasons behind the absence of IM Epi administration in some anaphylaxis cases within the delayed group remain ambiguous. Our primary hypothesis is that it is likely that patients possessed an epinephrine autoinjector, such as an EPIPEN®, at home and were advised by EMS staff to use it upon dispatcher's approval, thereby negating the need for MMT intervention with epinephrine. Secondly, the lower incidence of anaphylaxis cases in the delayed MMT group suggest that more severe instances were promptly identified following the initial call by the patient or a bystander leading to an immediate MMT dispatch.

In comparison to other nations where dispatcher decisions are guided by standardized protocols like the "Medical Priority Dispatch System", in French EMCC, the decision-making process relies primarily on the judgment of physicians, including cases of anaphylaxis or any other dyspnea case. This has been furthered explained in the setting paragraph of the methodology section for readers better understanding.

Page 11, line 215: there is a minor mistake in the result i %. It says "(9.8.0%). It should be corrected.
Authors response: Thank you, this has been corrected.

In your Table 2: can you comment on why Heparin IV is used almost twice as often in the "Delayed MMT"? Authors response: The most plausible explanation for this discrepancy is likely associated with the higher proportion of patients with a diagnosis of pulmonary embolism within this group.

In Fig. 2: You are mentioning 2,076 cases and analysis of 870 cases, but I cannot se this in the figure 2 flow chart.

Authors response: We have re-evaluated this number and identified a mistake in how the results were initially presented. The flowchart presented in Figure 2 has been updated to improve understanding of results.

In the discussion you might be able to compare to systems where the BLS unit administer B2-agonist, epinephrine, corticolsteroids and/ or PEEP, so the delay of the MMT (no matter the cause) might be less important from a patient safety perspective. If the BLS unit administered the medications mentioned above, could that have changed your patient outcome in the data set?

Authors response: Thank you for your input. Considering the limited prerogatives of BLS units within the French prehospital system, the delay in dispatching MMTs could be indeed more critical compared to other systems. This issue has been further explored in the discussion section.

You are considering video communication integration to the dispatcher. There are several scientific articles on that subject. I can recommend, that you read the articles from Linderoth et al:
<https://pubmed.ncbi.nlm.nih.gov/34509558/>
<https://pubmed.ncbi.nlm.nih.gov/34488626/>
<https://pubmed.ncbi.nlm.nih.gov/35670423/>

Authors response: Thank you for providing us with these insightful publications, which will be valuable for our future projects. One has been included in the discussion section of the present manuscript.
